# Factors that affect clinical youth engagement in digital mental health research: a qualitative sub-study nested within a prospective cohort study

**DOI:** 10.1186/s12874-025-02571-9

**Published:** 2025-04-30

**Authors:** Amanda Bye, Emma Wilson-Lemoine, Kylee Trevillion, Ben Carter, Rina Dutta

**Affiliations:** 1 Department of Child and Adolescent Psychiatry, Institute of Psychiatry, Psychology and Neuroscience, King’s College London, London, UK; 2 Department of Psychological Medicine, Institute of Psychiatry, Psychology and Neuroscience, King’s College London, London, UK; 3 Health Service and Population Research Department, Institute of Psychiatry, Psychology and Neuroscience, King’s College London, London, UK; 4 Department of Biostatistics and Health Informatics, Institute of Psychiatry, Psychology and Neuroscience, King’s College London, London, UK; 5 South London and Maudsley NHS Foundation Trust, London, UK

**Keywords:** Suicide & self-harm, Child & adolescent psychiatry, Mental health, Statistics & research methods, Qualitative research, Social media

## Abstract

**Background:**

There has been extensive debate about the role of social media and smartphone use in youth mental health and self-harm. Research to date lacks sufficient detail to determine the mechanisms underpinning any associations. The Social Media, Smartphone use and Self-harm in Young People (3S-YP) study is a prospective cohort study that was co-produced with young people to investigate temporal patterns of social media and smartphone use prior to an episode of self-harm in a clinical youth sample. Young people were actively involved in all key stages of the research process to ensure the research would be relevant and acceptable to the intended population. This included defining the research question and designing the methods. This qualitative sub-study nested within the main 3S-YP study aimed to evaluate young people’s experiences of engaging in this innovative digital mental health study. This will help inform understanding regarding the added value of co-production and future research in this field.

**Methods:**

Semi-structured interviews were conducted with a purposive sample of participants from the 3S-YP study. Interview data was analysed using codebook thematic analysis.

**Results:**

Sixteen young people (mean 19.8 years old, SD 2.9; *n* = 10 female, 63%) participated in the interviews. Participants were generally comfortable answering questions about sensitive topics using remote digital tools, appreciating the greater privacy, convenience and opportunity for self-reflection they provide, whilst noting periods of poor mental health may affect study engagement. The remote research methods (including the participation information and tools for recruitment and data collection) were considered user-friendly and were complemented by the active role of the research team who facilitated young people’s engagement with the study. Despite the relevance and support for research on the impact of digital technology use on youth mental health, concerns about data sharing and a complex process for accessing data from social media platforms complicated study engagement. The role of parental involvement was also described.

**Conclusions:**

User-friendly remote research methods, coupled with proactive, responsive researchers and parental support are beneficial for conducting research with clinical youth populations. Whilst young people endorse research in this field, concerns about data sharing and barriers to data access need addressing if researchers are to effectively employ innovative solutions to investigating the impact of smartphones and social media use on youth mental health and self-harm. The findings from this study demonstrate the value of actively involving those with lived experience throughout the research process and provide useful insight for researchers intending to conduct similar research.

**Trial registration:**

This study is registered on ClinicalTrials.gov (ID no. NCT04601220).

**Supplementary Information:**

The online version contains supplementary material available at 10.1186/s12874-025-02571-9.

## Background

With the rapid growth of digital technology over recent decades, smartphones and social media have become integral to daily life, especially among young people [1]. By the age of 12 years, most young people in the UK own a mobile phone and use it to spend increasing amounts of time online, including to access social media [2]. There has been ongoing speculation about the role of smartphone and social media use in youth mental health because since their expansion an unprecedented rise in mental health problems and self-harm among young people has been observed [3, 4]. The National Institute for Health and Care Excellence defines self-harm as ‘intentional self-poisoning or injury, irrespective of the apparent purpose’ [5] and is associated with an elevated risk of significant adverse outcomes, including mental illness and suicide [6, 7]. Youth mental health and self-harm are significant public health priorities so research to understand the underpinning mechanisms and moderating factors are of paramount importance.

Problematic smartphone use (PSU), i.e. dysfunctional usage, such as neglecting other activities due to excessive time spent using a smartphone, has been linked to an elevated risk of anxiety, depression and sleep disturbance [8], and there is emerging evidence implicating a possible association with self-harm [9]. Likewise, certain aspects of social media, including cyberbullying, exposure to harmful content (e.g. sharing and viewing self-harm content) and excessive and problematic usage, have been associated with self-harm and mental health problems [10, 11]. There is evidence, largely captured through qualitative investigations, that positive interactions on social media can also be beneficial for youth mental health, including providing opportunities for peer support and social connectedness that may be particularly beneficial for young people with mental health difficulties and those from marginalised communities [11, 12].

Overall, while there is some empirical evidence linking smartphone and social media use with self-harm and adverse mental health, studies have demonstrated small to medium effect sizes and are yet to establish the direction of an association or a causal link, or understand the role of other factors, with questions remaining regarding the quality of the evidence [10, 13]. This is largely due to the predominance of cross-sectional study designs (or large gaps between waves of data collection), inconsistencies in definitions and measurements, a reliance on retrospective self-report and the absence of linked survey data in the case of web scraping from social media platforms [8, 10, 13]. There is a clear need for well-designed prospective studies, linking survey data on self-harm and mental health with objective measures of smartphone and social media use, i.e. data generated passively by smartphone devices and on social media platforms [14]. Given the potential for these innovative methodological approaches to deepen our understanding of how digital technologies affect youth mental health and self-harm, it is imperative that such projects ensure active participant engagement. This necessity arises from the sensitive nature of the topics under investigation, the introduction of novel data linkages and the reliance on participants to manually provide their data, as exemplified by consented access to social media data [15, 16].

Researching participant engagement in novel study designs is imperative so we can understand and learn from the experiences of participants for future research. Other literature indicates that participating in studies measuring self-harm are unlikely to cause distress or trigger behaviours, although this may vary according to gender, level of risk and methods [17, 18]. Willingness to share smartphone and social media data for research purposes varies widely [19–24], with the disclosure of private information and consented access to data being of paramount concern. Less is currently known, however, about attitudes to sharing smartphone and social media data among clinical youth samples or in studies actively collecting these forms of data. Qualitative studies can uncover important insights, such as what kinds of smartphone and social media information participants are willing to share with researchers (e.g. metadata and public posts, rather than private messages), and can explore common concerns or misconceptions around data sharing [20, 21]. Studying participant engagement in digital mental health research more broadly can elucidate practical barriers and identify facilitators to sustained involvement, including specific aspects that can enhance acceptability, feasibility and value of a digital mental health study [25, 26].

The Social media, Smartphone use and Self-harm in Young People (3S-YP) study is a prospective cohort study that was co-produced with young people to investigate temporal patterns of social media and smartphone use prior to an episode of self-harm in a clinical youth sample. Young people with lived experience were actively involved in all key stages of the research process from the outset to ensure the research would be relevant and acceptable to the intended population. This included defining the research question and designing the methods. Full details on the study, including how young people were involved in the research, have been published elsewhere [14, 27]. This paper reports on findings from a concurrent qualitative sub-study nested within the main 3S-YP study. The aim of this qualitative study is to explore young people’s subjective experiences of engaging in digital mental health research by reflecting on their experiences of participating in the 3S-YP study (from recruitment through to study completion, including the use of remote digital tools for recruitment and data collection, their willingness to share social media and smartphone data and the practicalities of providing this data for research). The findings of which will help inform understanding regarding the added value of co-production and future research in this field.

## Methods

### Study design

This study employed a qualitative research design comprising semi-structured interviews with participants from the 3S-YP study. We have reported this study in accordance with the COREQ reporting guidelines [28].

###  3S-YP study

**Fig. 1:**
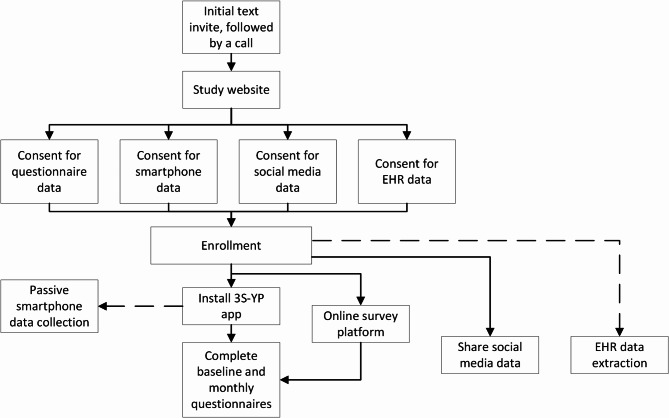
Process of participation

The 3S-YP study included participants aged between 13 and 25 years old recruited from the South London and Maudsley NHS Foundation Trust’s (SLaM) Consent for Contact (C4C) patient research participation register and who were followed up for 6 months [29]. Young people (or a parent and guardian for those aged 13–15 years old) received an initial text message (or email/telephone call), which researchers followed up a week later. Recipients were directed to the study website to access the written participant information, supplemented by a co-produced animation outlining what participation involves, and digital assent and consent forms. Separate consents were obtained for the study to collect questionnaire, electronic health record (EHR), social media and smartphone data. As a minimum, participants were required to provide questionnaire data. Those consenting to sharing smartphone data installed the co-designed 3S-YP app on their device - designed to extract metadata and administer monthly questionnaires during follow-up. Alternatively, an online survey platform (Qualtrics) was used to provide questionnaire data. Questionnaires covered sensitive topics, including self-harm, mental health and bullying victimisation. Participants received automated reminders (push notifications and/or text messages) inviting them to complete the monthly questionnaires. Consenting participants were invited to share their social media data at three time points, with researchers providing written and verbal instructions explaining how to access their data. Participants were able to choose which social media platforms they were willing to share their data from. EHR data for consenting participants was extracted after baseline and study completion. Figure 1 illustrates the process of enrolment and participation.

### Research team

The research team is multidisciplinary, with training in Epidemiology (EWL, RD), Health Policy (EWL), Health Sciences (AB, KT), Psychiatry (RD), Psychology (AB, EWL, KT) and Statistics (BC). Those involved in the analysis are experienced qualitative and participatory researchers (AB, EWL, KT, RD), including as a service user researcher (EWL). AB (PhD, King’s Maudsley Partnership for Children and Young People Translational Research Fellow, Project Manager for the 3S-YP study) recruited the sample, collected the data, and led and conducted the analysis, with EWL (PhD, Research Associate, not involved in data collection for the 3S-YP study). AB, EWL and RD (PhD, FRCPsych, Professor of Psychiatry and Academic Capacity Development, Consultant Psychiatrist and Chief Investigator for the 3S-YP study) met regularly throughout the analysis process to discuss and review the developing coding frame, with consideration of the varied disciplinary perspectives. KT (PhD, Deputy Director of the National Institute for Health and Care Research [NIHR] Policy Research Unit in Mental Health [MHPRU], Senior Lecturer, Qualitative Lead on the 3S-YP study) advised and contributed to the analysis discussions.

### Recruitment

Young people who had consented to be contacted for other studies (and a parent or guardian, for those aged 13–15 years) when they enrolled in the 3S-YP study were eligible to participate in this study. A purposive sample of young people were invited to participate in an interview once they had either completed the month-6 questionnaire or withdrawn from the 3S-YP study. We employed a maximum variation, purposive sampling strategy to recruit for the interviews, based on age, gender, ethnicity and engagement with the main study (for example, to include those who had experience of using the 3S-YP app and providing social media data), to gain insights into the disparate experiences of study participation across different groups [30]. Sample size sufficiency was guided by the concepts of data saturation [31, 32] and information power [33]. In terms of data saturation, we were satisfied with the final sample size as throughout data collection, we compared data arising from new interviews with previous interviews as well as across interviews to ascertain when new interview data no longer provided novel insights or significantly addressed the study aim [31, 32]. With regard to information power [33], given the scope of the study was relatively narrow and well defined, combined with a purposive sample of participants who reflected on their specific experiences of participating in the main study, we consider that the sample provided a sufficient understanding of the participants’ subjective experiences of study participation. Our knowledge of the main study and early development of a preliminary combined inductive/deductive coding frame, which was sufficiently supported as further interviews were coded, provided additional support for the final sample size and themes.

### Data collection

Semi-structured interviews were conducted via telephone at a time that was convenient to the young person. Interviews were guided by a comprehensive interview schedule that was co-designed with young people to generate discussions about the different study components. For example, ‘How did you find the experience of using the 3S-YP app?’, ‘What did you like about it?’ and ‘What do you think could be improved?’. This method ensured consistency across interviews whilst permitting the flexibility to probe based on a participant’s response [34]. Interviews were conducted by AB, which provided uniformity across the dataset and ensured the most important questions were posed and participants were given further detail where necessary based on the researcher’s in-depth knowledge of the 3S-YP study [34]. Participants gave informed consent via a digital consent form prior to the interview. Interviews took place between June 2022 and May 2023. The mean length of the interviews was 27 min and 20 s (SD 7 min and 59 s). Interviews were audio recorded with consent and transcribed. Participants were offered a £20 shopping voucher as a thank you for their time.

### Analysis

The data were analysed from a phenomenological theoretical perspective with emphasis on understanding the participant’s subjective experience of participating in the 3S-YP study [35]. We utilised a codebook thematic analysis approach [36], as has been effectively applied in other research [37–40]. This approach facilitated a collaborative and transparent coding process between researchers [36], the output of which will be useful for future work exploring participant experience among the wider study sample. Rather than to establish coding consensus, collaborative coding was valued for adding greater depth and facilitating a reflexive approach to the analysis, given the different levels of familiarity with the main study and the multidisciplinary perspectives of the research team [35]. The analysis followed six stages of thematic analysis as described by Braun and Clarke [41]: (1) familiarisation, (2) coding, (3) generating initial themes, (4) reviewing and developing themes, (5) refining, defining and naming themes, and (6) writing the report. Two researchers (AB, EWL) familiarised themselves with the data by reading each transcript independently and making notes on the salient details. The same researchers then independently coded two of the transcripts to generate an initial set of codes based on the data. Following which they met to discuss coding and develop a preliminary coding frame. The preliminary coding frame combined inductive and deductive approaches by incorporating the initial codes and themes identified from the subset of data alongside codes and themes informed by the interview schedule. AB and EWL then applied the preliminary coding frame to the rest of the transcripts, attending to all data in the coding process. The coding frame underwent several iterations to accommodate additional inductive codes that were created. Researchers documented their ideas and insights to complement the coding process and met regularly throughout to discuss and review the coding frame. Following the coding of all transcripts, the coding frame was finalised, and the themes were refined, defined and named. Data was managed in NVivo 14 [42].

## Results

### Sample descriptives

Sixteen young people participated in the semi-structured interviews, out of 35 participants (46%) who were invited to take part. The remainder did not respond to contact attempts (or in the case of one young person, declined to participate). This represents 5% (*n* = 339) of the wider 3S-YP sample who consented to be contacted for other studies. Table 1 summarises their demographic characteristics and participation data compared with all 3S-YP study participants. Compared to the 3S-YP cohort overall, the interviewed sample had a higher proportion of participants aged 16–17 years old, from an Asian or Asian British ethnic background, who were not in employment and were more engaged with the study (e.g. provided valid social media data). The sample was comparable in mean age in years and gender.

**Table 1:** Sample characteristics and participation data compared with the 3S-YP cohort

Characteristics	Interview sample (*N* = 16)		All 3S-YP study participants (*N* = 362)	
	*n* (%)	Mean (SD)	*n* (%)	Mean (SD)
Age in years		19.8 (2.9)		19.2 (3.2)
Age categories				
13–15 years old	-		39 (11)	
16–17 years old	5 (31)		79 (22)	
18–21 years old	7 (44)		155 (43)	
22–25 + years old	4 (25)		89 (25)	
Gender				
Female	10 (63)		225 (62)	
Male	5 (31)		106 (29)	
Prefer to self-describe	1 (6)		29 (8)	
Missing	-		2 (1)	
Ethnicity				
Any Asian or Asian British background	3 (19)		23 (6)	
Any Black, African, Caribbean or Black British background	3 (19)		51 (14)	
Any Mixed or Multiple ethnic background	2 (13)		64 (18)	
Any White background	8 (50)		212 (59)	
Any other ethnic group	-		10 (3)	
Missing	-		2 (1)	
Education status				
Secondary school	-		38 (11)	
Sixth form/college	6 (38)		103 (29)	
University	4 (25)		74 (20)	
Other (e.g. home tuition/pupil referral unit)	-		17 (5)	
None of the above	6 (38)		126 (35)	
Missing	-		4 (1)	
Employment status^a^				
Employed/self-employed	4 (25)		108 (30)	
Student	10 (63)		232 (64)	
Not working for health reasons	1 (6)		51 (14)	
Not in employment	4 (25)		33 (9)	
Other (e.g. carer/homemaker)	-		19 (5)	
Missing	-		10 (3)	
Highest level of education				
No formal qualifications	-		41 (11)	
GCSE grades 3 − 1/D-G, NVQ level 1 or equivalent	3 (19)		23 (6)	
GCSE grades 9 − 4/A*-C, NVQ level 2 or equivalent	5 (31)		111 (31)	
AS/A Level, NVQ level 3 or equivalent	6 (38)		112 (31)	
Diploma level or above	1 (6)		45 (13)	
Other	1 (6)		18 (5)	
Missing	-		12 (3)	
Consented to sharing smartphone data				
Yes	16 (100)		312 (86)	
No	-		50 (14)	
Installed the 3S-YP app				
Yes	15 (94)		287 (79)	
No	1 (6)		75 (21)	
Consented to sharing social media data				
Yes	15 (94)		283 (78)	
No	1 (6)		79 (22)	
Valid social media data available				
Yes	10 (63)		110 (30)	
No	6 (38)		252 (7)	

^a^Young people were able to select all response options that were relevant

### Themes

**Fig. 2:**
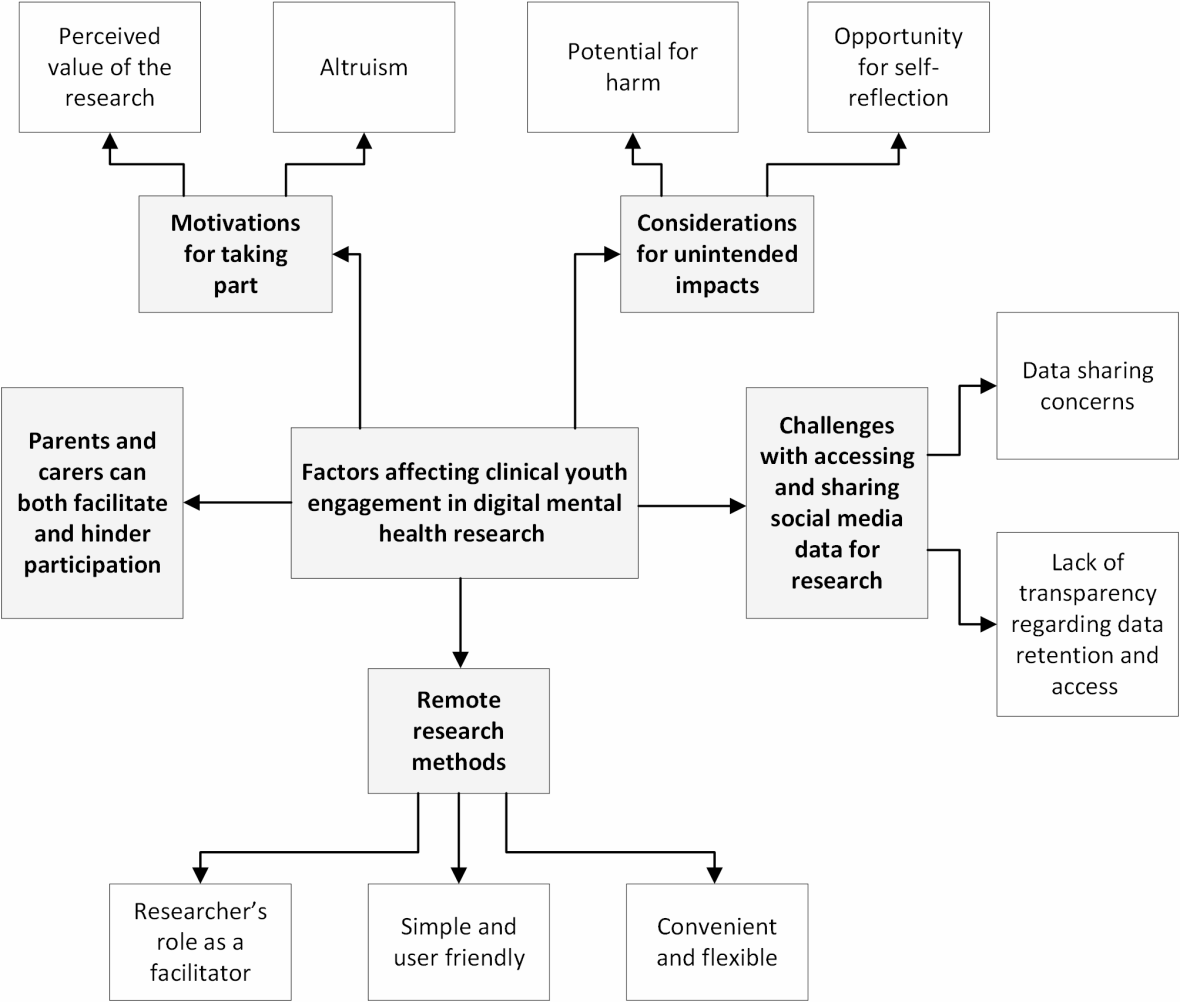
Conceptual map of factors affecting clinical youth engagement in digital mental health research

We identified five main themes regarding young people’s subjective experiences of engaging in digital mental health research. Themes are conceptualised in a thematic map (see Fig. 2). Quotations that are considered most illustrative of the themes are included in the results, with supplementary quotations presented in an additional table [see Additional file 1]. All quotations were reviewed to ensure participants could not be identified.

### Motivations for taking part

The first theme describes two key motivating factors - altruism and perceived value of the research – driving young people’s participation in digital mental health research.

#### Altruism

Many participants spoke of their interest in contributing to research for the benefit of other young people, as well as advancing our understanding of the links between digital technology use and youth mental health.

*“I’m very interested in taking part because it’s*,* uh*,* not only […] about your experience but also helping others and bettering others*,* uh*,* mental health*,* um*,* support systems” (Participant 13)*.

For a few participants, the benefits of the research, and their commitment to the advancement of research for the benefit of others, outweighed potential concerns or risks to themselves from taking part.

*“…if I weren’t in a good place*,* I might have got triggered*,* which is fine I would have still answered them.” (Participant 16)*.

#### Perceived value of the research

All participants endorsed the value of the study, reflecting on their personal experiences, particularly negative but also positive experiences of using smartphones and social media, and advocated for more research in the field.

*“…most people*,* especially young people are essentially attached to their phones. And it does*,* it does affect their mental health in some way*,* whether it be that they’re not sleeping enough or they’re always on their phone and they’re not connecting with the outside world […] there is a very clear link in day-to-day life between mental health and phones and social media.” (Participant 7)*.

Several participants expressed a need for a more nuanced understanding of the impact of digital technology use.

*“…it’s not really reflective sometimes because things like screen time*,* somebody may have a high screen time but that’s because they had their phone open playing music. So*,* it may come across as they were on their phone*,* but they weren’t. Whilst other people don’t necessarily go on their phone but when they do*,* they are constantly on social media” (Participant 11)*.

Related to this desire to contribute to meaningful research, some participants spoke about doubting their value as a participant during the study due to their low digital technology use and improved mental wellbeing.

*“…sometimes I felt like I was*,* like*,* I wasn’t really providing any*,* any actual information because I was always like ‘I’m sleeping fine*,* no one’s bullying me*,* um I*,* I don’t feel bad using social media’…” (Participant 1)*.

### Considerations for unintended impacts

The second theme describes potential unintended impacts of participating in digital mental health research, both negative and positive.

#### Potential for harm

Participants were generally comfortable with the sensitive topics covered in the questionnaires, reflecting on their experiences of discussing their mental health with health professionals and increasing self-awareness. They appreciated that the questions required only brief responses, rather than in-depth accounts of self-harm episodes that may be more distressing for an individual to recount.

*“I didn’t have to write like a very triggering paragraph […] and also because*,* like*,* in the service that I’m in […] I’m kind of used to questionnaires […] so yeah it was fine” (Participant 15)*.

However, several participants felt that the sensitive nature of the questions may have caused distress for those currently experiencing mental health symptoms. Also, participants who were experiencing fluctuating symptoms expressed difficulties with averaging their symptoms for the standardised measures.

*“…I might have felt one way over the last few weeks. But maybe in the last few days*,* or on that day I felt*,* um*,* differently […] your emotions can change*,* you know*,* quite frequently.” (Participant 9)*.

#### Opportunity for self-reflection

The ability to complete the questionnaires independently and on a regular basis led to participants self-reflecting on their mental health and use of digital technology. One young person described feeling less alone because their experiences were reflected in the questionnaire content.

*“I thought it was really beneficial for me really because it made me […] have a deep look about how things actually affect me when I’m not realising it.” (Participant 16)*.

### Parents and carers can both facilitate and hinder participation

The third theme addresses the role of parents and carers in the participation experience. For a few participants, it was their parent who was initially contacted by the researchers. More often this was received positively, particularly for those who were uncomfortable speaking directly with researchers.

*“…I don’t really like socialising. So doing it*,* like*,* getting it on the link*,* and just me consenting by myself […] feels a lot better than talking to the person” (Participant 10)*.

One participant described their parent encouraging them to enrol and another spoke of the benefits of having their parent remind them when they needed to provide data for the study.

*“I had someone reminding me to do it” (Participant 8)*.

Conversely, there were some instances where it was agreed early on during enrolment that the parent would be the point of contact and this agreement was not revisited later, which led to difficulties for some participants with getting the necessary study information relayed by their parent.

*“Personally*,* it didn’t go all too well just because my mum forgets sometimes to talk through about stuff that she gets emailed about or called about. Or just*,* sometimes I’m never there to also talk with…” (Participant 7)*.

### Challenges with accessing and sharing social media data for research

The fourth theme comprised of two sub-themes describing the challenges with accessing and sharing social media data with the study.

#### Data sharing concerns

Participants held similar views about novel data sharing for research, with more being apprehensive about sharing their social media data compared to the few who expressed concerns about sharing questionnaire data and smartphone metadata. Social media was described as a form of escapism for young people and thus researchers accessing their data for the study may feel like an invasion of privacy.

*“…it does seem a little bit intrusive*,* um*,* just because we use it as kind of a getaway*,* like an escape from*,* you know*,* stress of life” (Participant 14)*.

Participants who expressed fewer data sharing concerns did not think they posted much private information on social media, or they reflected that there will be organisations that already have access to their data.

*“…I think I’m pretty blasé about it because*,* uh*,* so many different things already have access to it.” (Participant 6)*.

There were some differences of opinion regarding acceptable parameters of social media data sharing for research. Participants were reassured that their data would be anonymised and were generally comfortable with sharing metadata, search history, data from platforms they use less or those that are more public facing (e.g. X, formerly Twitter), reactions and comments. They all expressed an unwillingness to share the content of private messages or data on friends or followers.

*“I was fine with the study accessing like what I’d liked or commented maybe. Um*,* I think*,* yeah*,* as you said*,* I was less*,* um*,* okay with […] the private messages” (Participant 3)*.

Given the potential for data sharing concerns, participants appreciated the flexible approach to participation. However, some felt that the ability to choose what data to share with the study could compromise the validity of the results as young people’s use of social media varies considerably between the different platforms available.

*“…it’s good that you personally*,* like*,* asked us if we wanted to provide it. And*,* like*,* you can choose what social media to choose from […] I use*,* like*,* Twitter […] and I’m looking at other people’s reactions to certain bits in the episode […] But that would be completely different from how I use Instagram where I’m talking to friends […] if I got*,* I can choose what apps I wanted*,* the data would be completely different from different apps. So […] it’d be a bit biased depending on what app I choose” (Participant 7)*.

#### Lack of transparency regarding data retention and access

Participants reflected that before taking part in the study, they were unaware what data is stored by social media platforms and how they can access their data.

*“…they do keep a lot more information than you actually do think they would have” (Participant 10)*.

*“…I had no idea how you could like download all of your information.” (Participant 1)*.

Participants who attempted to share their social media with the study, described a complicated process for accessing their data from the social media platforms. Some participants were more able to understand the process of providing their social media data than others, and this tended to be those who were older, had improved mental health or some form of computer training, though all required technical assistance to some degree. Even after several attempts, some remained unable to access their data.

*“…it was quite easy once I knew how to do it*,* really…” (Participant 16)*.

*“I don’t think I actually provided any because I found the process*,* like*,* quite hard to do it […] I tried to request from Instagram*,* and it just did not work” (Participant 7)*.

Participants found the process inconvenient as there is a delay after submitting a request to the platforms before a user can access their data and users are given a short window of time to download their data before the web link to the data file expires.

*“…it was a bit annoying because it’s not like […] you click on it*,* you download it […] you wait for the […] email link and you can download it within […] 24 hours*,* 48 hours or whatever. And then if you miss that*,* you have to go through the whole thing again” (Participant 15)*.

### Remote research methods

The final theme comprised three sub-themes summarising the young people’s experiences of engaging with the remote research methods employed in the study.

#### Researcher’s role to facilitate

Participants described how the researchers facilitated their participation in the study by providing further clarification and technical support where needed, as well as gentle reminders to provide data. Beyond these more practical aspects, the researchers also provided a much-valued human element and legitimacy to the remote research participation experience. Participants appreciated their friendly and flexible approach to data requests, as well as their proactive and responsive manner to participants’ needs.

*“…the team was so nice and just so easy to talk to […] even though a lot of it was online*,* there was […] a really big*,* like*,* human presence […] I knew that I could just call someone or text someone. I think that’s*,* that’s always good just to know that you can put like the*,* the survey to a*,* a voice*,* to someone that you know…” (Participant 1)*.

*“…it was really helpful that you guys were*,* you know*,* sending texts and following up with phone calls […] checking that everything was alright […] I didn’t feel pestered or anything but at the same time I didn’t feel completely on my own.” (Participant 5)*.

#### Simple and user friendly

Participants consistently described the remote research methods as *“simple”* and *“easy”* to use. The text message approach provided participants with the opportunity to learn about the study before speaking with a researcher. Although it is worth noting a few young people voiced having initial concerns about the legitimacy of the text message, with it being from an unknown telephone number.

*“…it gave me the information I needed*,* yeah*,* and stuff I needed to know without necessarily having to call” (Participant 4)*.

The participant information was considered acceptable and accessible, with some participants expressing that the video was a useful alternative or supplement to the written information.

*“…everything was easy to read and there was no*,* um*,* complex words or anything too difficult…” (Participant 2)*.

Several participants felt there was a need for more detail regarding the less familiar and more complex aspects of the study, including the need to rephrase certain terminology.

*“…when I personally had heard the phrase smartphone data*,* I just think you were going to hack into my phone*,* collect any data you could find…” (Participant 7)*.

The study website and app were considered accessible and visually appealing, though there were some differences of opinion regarding font size on smartphone apps, with participants generally able to navigate them without needing much, if any, technical assistance.

*“It was nice*,* easy to use. Um*,* the format was*,* um*,* very appealing. Um*,* nice colours and stuff and quite simple to understand” (Participant 4)*.

Participants spoke of several helpful app functionalities - notifications when a questionnaire was due (without which many would have “*forgotten*”), forewarning of sensitive items and access to resources for support. Although the simplicity was highly valued, a visual schedule, individualised feedback on data provided and the ability to upload social media data to the app would have enhanced the participation experience.

*“…there was always*,* like*,* a message to say*,* like*,* ‘these questions might be a bit difficult […] at the end there would always be […] the website you can go to*,* the phone numbers you can call. So*,* I think it was handled as best as*,* as it could have been handled” (Participant 1)*.

*“…if there were a log of like you haven’t done the thing for the month*,* that might have helped. Cos [sic] I think there was one time where I wasn’t sure if I’d done it or not…” (Participant 15)*.

#### Convenient and flexible

Young people felt that participating in remote studies was convenient, particularly for those with caring responsibilities or those who did not live locally, and given the longitudinal nature of the study.

*“…it was just quite convenient to do it online because you didn’t have to set aside too much time or any*,* you know*,* time travelling to or from one place” (Participant 9)*.

Participants felt that the use of remote digital tools for recruitment and data collection allowed them more time and space to consider their answers independently. For these reasons, some participants felt that remote research methods may be more acceptable for neurodiverse individuals.

*“…there was more space for me to take it at my own pace rather than feeling any sort of pressure from like somebody actually looking at me […] I felt a lot more secure about my decision to proceed in the study…” (Participant 6)*.

There were a few participants who expressed a preference (or indifference) towards in-person or telephone research visits, suggesting for some the benefits of remote research methods may be outweighed by the benefits of social interaction.

*“…I would have preferred it to be face-to-face*,* uh*,* just for personal reasons I find it more*,* more*,* uh*,* easier to*,* uh*,* talk to someone” (Participant 13)*.

## Discussion

### Summary of the findings

In the present study, we used semi-structured interviews to identify factors that affect clinical youth engagement in a digital mental health study exploring social media and smartphone use and self-harm. Our sample reflected on the whole process of engaging in a remote study from recruitment through to study completion, including the use of remote digital tools for recruitment and data collection, their willingness to share social media and smartphone data and the practicalities of providing this data for research. Overall, we found that participants were generally comfortable providing limited information regarding sensitive issues such as self-harm, mental health and bullying victimisation using remote digital tools, though periods of poor mental health may affect study engagement. Participants reported altruistic reasons for participation, appreciating opportunities for self-reflection on their mental health and technology use. Participants advocated for research into the topic of digital technology use on mental health, whilst also voicing concerns about sharing social media data (particularly private messaging or data on friends or followers) and encountered complexities in accessing data from social media platforms. Our results also highlight the importance of the people around the young person, with parents and carers often having a key role in facilitating their young person’s involvement in research. Proactive, responsive researchers serve a dual purpose of supporting with queries, particularly for participants who may need technical support, and building sufficient trust with participants to facilitate participation. Finally, the remote research methods employed in the study were considered user friendly and allowed for greater privacy and convenience than in-person data collection methods, though this simplicity may have come at the expense of providing more in-depth detail regarding the less familiar, more complex aspects of study participation.

### Comparisons with previous research

We found that young people were motivated to participate in the main study due to altruism and an interest in the research topic. Altruism has consistently been identified as a key motivation for participating in health research, with young people wanting to contribute to research that will benefit other young people in the future who share similar experiences [18, 21, 43]. Although, in line with other research [43], we found that perceiving one’s contribution as unhelpful due to feeling well at the time of the study, may be a barrier to study participation. Our participants felt that research to investigate the mental health impacts of smartphone and social media use was important and relevant, in line with findings from previous priority-setting work in youth mental health research [44, 45]. A focus group with nine young people drawn from the Avon Longitudinal Study of Parents and Children (ALSPAC) found that they were keen, in principle, to help with research on this topic and this was related to their trust of ALSPAC researchers [21]. As will be discussed later, this underpins the important role of the research team in participant engagement.

As found in previous research [17, 18], measuring self-harm repeatedly did not cause distress (with participants in this study briefed about how they could skip questions and access resources for support), which is an important ethical consideration when designing self-harm studies. Through their experience accessing clinical services, participants were familiar with discussing these sensitive topics and appreciated the opportunity to develop their self-awareness, as has been highlighted in another longitudinal study about self-harm [18]. However, participants did report that poor mental health could act as a barrier to young people engaging with research. This concurred with a review of retention rates among digital mental health intervention studies [46], which found that despite a willingness for many young people to participate, mental and physical health stressors may negatively influence engagement. It seems appropriate, therefore, that digital mental health studies are flexible and accommodating of fluctuations in participant health. For example, participants in our study appreciated neither feeling pressured to take part nor judged, even if they missed a follow up questionnaire.

Our findings highlight the role of parents and carers in studies involving young people. A qualitative systematic review identified direct support from family, friends or peers can be a barrier or facilitator to successful recruitment or engagement into studies exploring digital health interventions [47]. A similar finding was found in a study of 16–19-year-olds with chronic health conditions [43], with the authors identifying parents as barriers or facilitators for participation. While parents and carers are legally required to consent for under 16s to participate in UK-based-research studies, it is notable that a parent or carer’s details were recorded as the primary contact on the EHR for many young people over 16 who were approached for the main study. For digital mental health studies with remote recruitment processes, parental buy-in is even more important, and researchers need to be well-equipped to present information about the study to address potential misunderstandings or concerns, particularly around risk and data privacy. This is important as there may be generational differences in attitudes towards data sharing [21].

Participants described the importance of anonymity when deciding whether to share their data for research, and felt more comfortable sharing smartphone and social media metadata (e.g. number of ‘likes’, number of followers and friends) than the content of private messages. This corresponds with previous findings, with greater willingness to share data that could be distilled to numbers and data points, than photos or location data, due to risk of losing anonymity [21]. Other studies have found that females, who comprise the majority of our sample, may hold more social media privacy concerns than males, which may partly be explained by their increased tendency to share more personal information (e.g. photos, status updates [48]). The desire for anonymity may be even greater in a study exploring self-harm, as some participants may interact with pro-self-harm online communities or content. For example, in a study exploring associations between eating disorders and social media, many participants refused to answer a question about accessing negative online content related to their eating disorder, with authors postulating it was due to fear of whether it will be shared with their parents or clinicians [49]. Likewise marginalised communities may be more cautious about sharing their data if for example, an individual’s information being made available could put them at risk [23]. Disparities in data completeness across different groups, based on factors such as socio-demographics and self-harm status, may compromise the validity of the findings and implications. Among participants with experience of psychosis, it is also important to consider whether they may have concerns that passive data tracking may exacerbate their symptoms of paranoia [50].

Despite our sample being relatively technologically able, many participants described the complexity around accessing their data from social media platforms, alongside a lack of transparency around data retention. This may lead to young people feeling as though they have limited control over the way their data is handled, emphasising a need for research to consider how to inform and empower young people to be data literate and have agency with regard to how their data is used [51]. These are important factors, with previous literature identifying participant burden associated with technical complexity as a barrier for participation and engagement with digital health studies [47, 52]. Moreover, participants increasingly expect systems within research to be integrated and automated, with previously negative experiences dissuading their decision to participate in future studies using technology [47]. In a recent commentary piece, we highlighted the barriers imposed by social media platforms that largely preclude researchers from accessing social media data, even with clearly documented informed user consent, and proposed a framework to facilitate regulated and monitored consented access for researchers [16]. Moreover, Wellcome and the UK Medical Research Council (MRC) advocate for greater data linkage between existing datasets and social media platforms [21, 53, 54], with several active (and proposed) projects underway using UK longitudinal cohorts, though limited to specific platforms that are less utilised by younger generations [55, 56]. With participant consent, these proposals may alleviate barriers to participation in studies exploring social media and smartphone use, however, despite the necessity, engagement from social media companies is not yet forthcoming [16]. We urge policymakers to mandate social media platforms to make it easier for consenting research participants to access their social media data.

Among the practical aspects of taking part in the 3S-YP study, participants valued the accessibility of the remote digital tools for enrolling in the study and completing questionnaires. Technical literacy of the intended population is an important factor for researchers to consider when designing digital mental health studies. This has previously been explored in healthcare when considering uptake of digital technologies, with concerns of data poverty and exacerbating well-documented income and racial inequalities [52, 57]. Given the existing health challenges faced by participants recruited from clinical settings, additional efforts must be made to bolster confidence and ability to engage with technology, proactively offering on-hand technical support, while ensuring the remote research methods are as simple as possible through co-design and user testing as was the case with 3S-YP study. In line with previous research [47], participants appreciated the ability to choose the time and location of study participation, as well as the added independence and privacy that remote participation offers [47]. Moreover, similar to the wider digital health literature [26, 46], young people valued features such as personalisation, limited text and discrete push notifications or text message reminders for completing tasks. These are important considerations, given that young people may be navigating a host of typical life transitions, such as leaving school, moving out from the family home, or starting college, university, training or employment, and specific to mental health service users, moving from child and adolescent into adult services.

The descriptions of the research team as responsive, well-informed and providing reassurance are other crucial aspects of encouraging initial and sustained involvement in a remote study, particularly one that involves the novel sharing of social media and smartphone data. Drawing on a systematic review exploring barriers and facilitators to research involvement in digital mental health studies [47], recruitment challenges included difficulties understanding the recruitment message, while facilitators included active promotion and engagement strategies. Alongside the importance of clear participant information, our findings demonstrate the need for researchers to provide prompt follow-up after initial approach to clarify the scope of the study. Moreover, another study similarly identified research team support as facilitating continued engagement with a study using remote monitoring technology [26]. This included how to use the study app and alleviating any initial data privacy concerns, as well as researchers who were approachable, patient and responsive. From our study, effective communication from researchers played an essential role in reassuring participants about how their data will – and will not – be used, building trust, which in turn encouraged participants to share their data, or alternatively reminded them of the freedom to change their mind regarding data sharing.

A key impact of the findings discussed is the value of co-production of research with young people, defined as shared power and responsibility to create equal partnerships between experts by experience and researchers, from study inception through to dissemination [14, 58, 59]. Given the pace of change and differential patterns of social media and smartphone use between generations, and the sensitivity of a topic such as self-harm, meaningful youth engagement is crucial for ensuring a study’s acceptability, feasibility and relevance [60]. Indeed, we effectively embedded co-production in the 3S-YP study from the outset, as evidenced by the overall acceptability of the study and participation experience among interviewees.

### Strengths and limitations of this study

A major strength of this study is that interviews were undertaken with a subset of participants from the 3S-YP study, which to our knowledge is the first of its kind to link survey and EHR data with objective measures of digital technology use in a clinical youth sample, thus offering a unique and efficient means of studying participants’ experiences of this novel methodological approach. The present study furthers current understanding about the public’s willingness to share their smartphone and social data for research [19–24], with participants able to reflect on actual rather than hypothetical experiences of providing such data and from a range of social media platforms. And specifically, young people who have accessed secondary mental health services, a group who warrant more attention in the literature given their increased risk of experiencing online harms. We employed a rigorous and transparent approach to data collection and data analysis [40, 61]. Finally, through effective engagement with youth experts by experience in the design of this (as well as the wider) study, we ensured our interview schedule was acceptable and appropriate, which undoubtedly enriched the interviews and resulting findings.

Despite the novelty of our study, there are limitations that warrant consideration. Not all young people who were approached took part in this study and interviews were not conducted with those who withdrew from the main study or were below 16 years old. It is possible that the experiences shared by young people in this study differ from those who were less engaged in the research or whose study participation required greater parental input. However, there were ethical and practical challenges to consider [62], including the need to interview those with experience of the different aspects of participation so we could address the study aim. There is an underrepresentation of males, consistent with the wider 3S-YP study, that should be considered when interpreting the results given the potential differences in attitudes to data privacy and data sharing [20, 21, 48]. Interviews were conducted by AB who was not independent of the 3S-YP study, and this may have had implications for this sub-study. Further, there is a risk of selection bias as interviews were conducted in English and the C4C population from whom the 3S-YP sample were identified, may not reflect the wider SLaM patient population [63].

## Conclusion

This study demonstrates the benefits of user-friendly co-designed remote methods for conducting research with clinical youth populations and the importance of empowering youth to make informed decisions about data sharing, emphasising their freedom to change their mind about how they engage with research. Our results also highlight the importance of parental support and proactive, responsive researchers. Concerns about data sharing and barriers to data access need addressing if researchers are to effectively draw on youth support and employ innovative solutions to investigate the links between smartphones and social media use on youth mental health and self-harm. Embedding co-production and evaluation in innovative research is imperative so that we can learn from and incorporate the experiences and perspectives of the intended population in the development of research, and through this study, we hope to offer rich insight for researchers aiming to conduct similar studies.

## Electronic supplementary material

Below is the link to the electronic supplementary material.

Supplementary Material 1

## Data Availability

Due to conditions of participant consent, and as it contains potentially sensitive and identifiable information, the data supporting this article cannot be openly shared. To request access, email research.data@kcl.ac.uk. A descriptive record can be found in the King’s College London research data repository, KORDS, at DOI: 10.18742/25043606.

## Abbreviations

C4C: Consent for Contact
EHR: Electronic health records
PSU: Problematic smartphone use
SLaM: South London and Maudsley NHS Foundation Trust’s
3S-YP study: Social media, Smartphone use and Self-harm in Young People study
