# Supplementary material for: Factors that affect clinical youth engagement in digital mental health research: a qualitative sub-study nested within a prospective cohort study
Source: BMC Med Res Methodol. 2025 Apr 30;25:118. doi: 10.1186/s12874-025-02571-9 (PMC12042430; doi:10.1186/s12874-025-02571-9)
Supplement: Supplementary file 1 — Supplementary Material 1 [file 12874_2025_2571_MOESM1_ESM.docx]

**Additional File 1: Description and illustrative quotations for themes 1 to 5**

| **Theme** | **Sub-theme** | **Description** | **Example quote** |
| --- | --- | --- | --- |
| Motivations for taking part |  |  |  |
|  | Altruism | Participants described wanting to help other young people by contributing their experience to research. For a few, their desire to contribute to research for the benefit of others, outweighed potential concerns or risks to themselves from taking part. | *“I'm very interested in taking part because it's, uh, not only […] about your experience but also helping others and bettering others, uh, mental health, um, support systems” (Participant 13)*    *“...if I weren’t in a good place, I might have got triggered, which is fine I would have still answered them.” (Participant 16)* |
|  | Perceived value of the research | Participants endorsed the aims of the study, stressing more nuanced research was needed to understand the impact of digital technology use on mental health, particularly for the benefit of young people with mental health difficulties. Related to this, some participants doubted the value of their data due to their low technology use and mental health symptoms. | *“…most people, especially young people are essentially attached to their phones. And it does, it does affect their mental health in some way, whether it be that they’re not sleeping enough or they’re always on their phone and they’re not connecting with the outside world […] there is a very clear link in day-to-day life between mental health and phones and social media.” (Participant 7)*  *“…it’s not really reflective sometimes because things like screen time, somebody may have a high screen time but that’s because they had their phone open playing music. So, it may come across as they were on their phone, but they weren’t. Whilst other people don’t necessarily go on their phone but when they do, they are constantly on social media” (Participant 11)*  *“…sometimes I felt like I was, like, I wasn’t really providing any, any actual information because I was always like ‘I’m sleeping fine, no one’s bullying me, um I, I don’t feel bad using social media’…” (Participant 1)* |
| Considerations for unintended impacts |  |  |  |
|  | Potential for harm | Participants were generally familiar and comfortable with answering questions on sensitive topics, although their current mental health may impede their ability to participate. | *“I didn’t have to write like a very triggering paragraph […] and also because, like, in the service that I’m in […] I’m kind of used to questionnaires […] so yeah it was fine” (Participant 15)*  *“I feel like it really just depended on how I was feeling on the day that I took them” (Participant 7)*  *“I’m in a much better place now so I was able to answer them. But I feel like maybe if I weren’t in a good place, I might have got triggered…” (Participant 16)*  *“…I might have felt one way over the last few weeks. But maybe in the last few days, or on that day I felt, um, differently […] your emotions can change, you know, quite frequently.” (Participant 9)* |
|  | Opportunity for self-reflection | Participants expressed that participating in the study led to self-reflection on their mental health and digital technology use. | *“I thought it was really beneficial for me really because it made me […] have a deep look about how things actually affect me when I’m not realising it.” (Participant 16)*  *“I just, kind of, got a bit more conscious of how much I was using it, like, I was paying more attention to my, like, weekly screentime notifications” (Participant 15)*  *“…made me feel like I’m not alone and that there are other people who go through the same kind of thing...” (Participant 8)* |
| Parents and carers can both facilitate and hinder participation |  | Participants described the benefits of their parent facilitating the enrolment and participation process, including instances where young people may be uncomfortable speaking directly with researchers. Conversely, some participants described issues when study information was relayed to their parent rather than with them directly. | *“…I don’t really like socialising. So doing it, like, getting it on the link, and just me consenting by myself […] feels a lot better than talking to the person” (Participant 10)*  *“…my mum just said she thought I should do it…” (Participant 12)*    *“I had someone reminding me to do it” (Participant 8)*  *“Personally, it didn’t go all too well just because my mum forgets sometimes to talk through about stuff that she gets emailed about or called about. Or just, sometimes I’m never there to also talk with…” (Participant 7)* |
| Challenges with accessing and sharing social media data for research |  |  |  |
|  | Data sharing concerns | Participants described what they perceived to be acceptable levels of data sharing, with sharing social media data being the most contentious. This was in stark contrast with the sharing of questionnaire data and smartphone metadata, which most were comfortable with. There were some discrepant viewpoints regarding the types of social media data that were considered acceptable, but all were uncomfortable with sharing the content of private messages and data on friends and followers. Given the potential for data sharing concerns, participants greatly valued the flexible approach to participation and the ability to choose what data to share with the study, however it was acknowledged this could compromise data validity. | *“…I’m quite open with all my data on my phone in the sense of anything that doesn’t require privacy. So, I was fairly unphased…” (Participant 12)*  *“I felt […] not that it was an invasive thing. I felt more embarrassed because I am aware that I spend quite a lot of time on my phone” (Participant 6)*  *“…it does seem a little bit intrusive, um, just because we use it as kind of a getaway, like an escape from, you know, stress of life” (Participant 14)*  *“…I think I’m pretty blasé about it because, uh, so many different things already have access to it.” (Participant 6)*  *“I’m happy to share, uh, the number of messages that I sent […] how many friends I had or followers I had” (Participant 9)*  *“I was fine with the study accessing like what I’d liked or commented maybe. Um, I think, yeah, as you said, I was less, um, okay with [...] the private messages” (Participant 3)*  *“…my friends and I, we talk about our mental health together […] I was worried that, um, if some, like, system, like, scanned our text messages or whatever, and picked up, like, self-harm or something and then, like, went through them and flagged it up, and then, like, they got in trouble or something” (Participant 15)*  *“…it’s good that you personally, like, asked us if we wanted to provide it. And, like, you can choose what social media to choose from […] I use, like, Twitter […] and I’m looking at other people’s reactions to certain bits in the episode […] But that would be completely different from how I use Instagram where I’m talking to friends […] if I got, I can choose what apps I wanted, the data would be completely different from different apps. So […] it’d be a bit biased depending on what app I choose” (Participant 7)* |
|  | Lack of transparency regarding data retention and access | Participants described a lack of transparency on what data is stored by social media platforms and how they can access their user data. Participants described the process of accessing their social media data as complicated, with delayed data access and time-limited data download links. | *“…they do keep a lot more information than you actually do think they would have” (Participant 10)*  *“I didn’t know you could access your data for a start. I didn’t know it was possible to […] have a look at everything that you’ve done...” (Participant 5)*  *“…I had no idea how you could like download all of your information.” (Participant 1)*  *“…it was quite easy once I knew how to do it, really…” (Participant 16)*  *“It was a bit confusing. Like, I found the [study] instructions really clear but just the actual, kind of, social media platform itself was just a bit of a nightmare…” (Participant 5)*  *“I don’t think I actually provided any because I found the process, like, quite hard to do it […] I tried to request from Instagram, and it just did not work” (Participant 7)*  *“…it was a bit annoying because it’s not like […] you click on it, you download it […] you wait for the […] email link and you can download it within […] 24 hours, 48 hours or whatever. And then if you miss that, you have to go through the whole thing again” (Participant 15)* |
| Remote research methods |  |  |  |
|  | Researcher’s role to facilitate | Participants described how the researchers facilitated their participation in the study by providing further clarification, technical support and reminders for data. Further, the researchers provided a human element and legitimacy to the remote research experience, with their friendly and relaxed approach to data requests, as well as their proactive and responsive manner to participants’ needs. | *“At first, I was a bit confused as to how to do it […] I then got some support in terms of how to access the data and all that kind of stuff, and then I found it, kind of, straightforward from there…” (Participant 12)*  *“…the team was so nice and just so easy to talk to […] even though a lot of it was online, there was […] a really big, like, human presence […] I knew that I could just call someone or text someone. I think that’s, that’s always good just to know that you can put like the, the survey to a, a voice, to someone that you know…” (Participant 1)*  *“…it was really helpful that you guys were, you know, sending texts and following up with phone calls […] checking that everything was alright […] I didn’t feel pestered or anything but at the same time I didn’t feel completely on my own.” (Participant 5)*  *“I found it quite helpful, kind of, cos [sic] I’m quite a forgetful person […] I don’t think I managed to do every single, um, questionnaire” (Participant 15)*  *“…whenever I needed to ask a question there was always someone there to help me and they got back to me really fast” (Participant 16)* |
|  | Simple and user friendly | Participants described the remote research methods as “simple”, “easy” and accessible. The initial text message approach was efficient, although a few had concerns about its legitimacy. The written information was acceptable, with some participants valuing the provision of a video outlining the study as an alternative or in addition to the written information. However, further detail was needed for the less familiar and more complex aspects of participation, i.e., terminology to describe smartphone metadata and the process for sharing social media data and what this data included. The website and app were user friendly and visually appealing. Advanced functionality could have enhanced the user experience. | *“…it gave me the information I needed, yeah, and stuff I needed to know without necessarily having to call” (Participant 4)*  *“…when you get an email from someone you don’t know these days it’s, like, is this a phishing scam […] is my information going to be stolen?” (Participant 1)*  *“…everything was easy to read and there was no, um, complex words or anything too difficult…” (Participant 2)*  *“…when things are, kind of, just written […] you may not understand it. But in the video, it was explained very, kind of, clearly and I feel like most people would understand what they were signing up for” (Participant 11)*  *“…when I personally had heard the phrase smartphone data, I just think you were going to hack into my phone, collect any data you could find…” (Participant 7)*  *“…very easy to use. Didn’t require a lot of you know tech skills or handling, neither of which I have” (Participant 5)*  *“It was nice, easy to use. Um, the format was, um, very appealing. Um, nice colours and stuff and quite simple to understand” (Participant 4)*  *“I also liked how quick and easy it was to complete. There wasn’t like an excess of either writing tasks or, um, extended set-up or communication that I had to go through” (Participant 6)*  *“…there was always, like, a message to say, like, ‘these questions might be a bit difficult […] at the end there would always be […] the website you can go to, the phone numbers you can call. So, I think it was handled as best as, as it could have been handled” (Participant 1)*  *“…if there were a log of like you haven’t done the thing for the month, that might have helped. Cos [sic] I think there was one time where I wasn’t sure if I’d done it or not…” (Participant 15)* |
|  | Convenient and flexible | Participants described remote methods as more convenient and flexible. Participants also expressed that remote research methods provided greater privacy and independence and were more acceptable for neurodiverse individuals. For a few, this may be outweighed by the benefits of social interaction. | *“…it was just quite convenient to do it online because you didn’t have to set aside too much time or any, you know, time travelling to or from one place” (Participant 9)*  *“…because it’s just one a month, it’s easy to keep track of and you don’t have to move around your schedule for it” (Participant 8)*  *“…there was more space for me to take it at my own pace rather than feeling any sort of pressure from like somebody actually looking at me […] I felt a lot more secure about my decision to proceed in the study...” (Participant 6)*  *“…say it was like a face-to-face appointment, I just feel like, I would feel inclined to answer a certain way […] Whereas this, it’s a database, it’s, I don’t feel inclined to answer in any certain way” (Participant 7)*  *“…is good for autism cos [sic] it’s difficult sometimes doing stuff like this…” (Participant 4)*  *“…struggle sometimes with maintaining eye contact…” (Participant 2)*  *“…I would have preferred it to be face-to-face, uh, just for personal reasons I find it more, more, uh, easier to, uh, talk to someone” (Participant 13)*  *“…the benefits of, like, a phone call conversation is that you go into perhaps a bit more depth with your answer rather than just an agree, strongly agree or disagree, kind of, scale or spectrum” (Participant 12)* |
